# Trait anger and negative interpretation bias in neutral face perception

**DOI:** 10.3389/fpsyg.2023.1086784

**Published:** 2023-05-05

**Authors:** Pauline Rohrbeck, Anette Kersting, Thomas Suslow

**Affiliations:** Department of Psychosomatic Medicine and Psychotherapy, University of Leipzig Medical Center, Leipzig, Germany

**Keywords:** trait anger, anger expression, facial emotion, schematic faces, negative attribution, hostile interpretation, anxiety, depressed mood

## Abstract

**Introduction:**

Anger is a basic emotion helping people to achieve goals by preparing the body for action and prompting others to change their behavior but is also associated with health issues and risks. Trait anger, the disposition to experience angry feelings, goes along with an attribution of hostile traits to others. Negative distortions in the interpretation of social information have also been observed in anxiety and depression. The present study examined the associations between components of anger and negative interpretation tendencies in the perception of ambiguous and neutral schematic faces controlling for anxiety, depressed mood, and other variables.

**Methods:**

A sample of 150 young adults performed a computer-based perception of facial expressions task and completed the State–Trait Anger Expression Inventory (STAXI-2) along with other self-report measures and tests.

**Results:**

Trait anger and anger expression correlated with the perception of negative affects in neutral but not in ambiguous faces. More specifically, trait anger was linked to the attribution of anger, sadness, and anxiety to neutral faces. Trait anger predicted perceived negative affects in neutral faces when adjusting for anxiety, depression, and state anger.

**Discussion:**

For neutral schematic faces, the present data support an association between trait anger and negatively biased interpretation of facial expression, which is independent of anxiety and depressed mood. The negative interpretation of neutral schematic faces in trait angry individuals seems not only to comprise the attribution of anger but also of negative emotions signaling weakness. Neutral schematic facial expressions might be useful stimuli in the future study of anger-related interpretation biases.

## Introduction

Triggered by frustration, unfair treatment, harm, or provocation, anger is commonly experienced by humans (Shaver et al., 1987). Its role as a survival response adapting the body to threatening situations makes it essential to our daily life. Being involved in the fight-or-flight response, it releases stress hormones such as adrenaline, testosterone, and cortisol, which increase the heart rate, respiration, body temperature, blood pressure, and muscle tone (Alia-Klein et al., 2020) so that the body is prepared for physical exertion. Socially, a beneficial effect of anger lies in helping people to achieve their goals by forcing others to change their behavior (Fischer and Roseman, 2007). Anger is considered one of the basic emotions humans experience across different cultures (Ekman, 1992). The facial expression of anger is typically characterized by staring eyes, eyebrows with lowered inner corners, and tightly pressed lips (Izard, 1977). Several studies highlighted a cross-cultural decoding of highly intense emotions in facial expressions (Ekman, 1994; Elfenbein and Ambady, 2002; Hess and Thibault, 2009). However, the explicit recognition of emotions in faces does not always succeed, in particular in case of the negative emotions fear, sadness, and anger (Kosonogov and Titova, 2019; Surcinelli et al., 2022).

According to Spielberger (1988), the experience of anger has two components: state and trait anger. State anger is described as an emotional condition characterized by individual feelings varying from moderate irritation to strong rage and usually combined with muscular tension as well as activated autonomic nervous systems (Spielberger et al., 1983). Anger as a trait refers to individual differences in perceiving situations as frustrating or dissatisfying and to the disposition to react with increased state anger (Spielberger et al., 1983). High trait anger goes along with more frequent and more intense experiences of state anger (Spielberger, 1988). Three types of anger expression have been distinguished: *anger expression-in* which represents the disposition to suppress anger or to redirect it to the self; *anger expression-out* which refers to the communication of anger toward the environment; and *anger-control* which is characterized by the effort to manage anger and to express the feeling with respect to the rights and emotions of the addressed person (*cf.* Spielberger, 1988). The State–Trait Anger Expression Inventory (STAXI-2; Spielberger, 1999), a self-report questionnaire, is the most commonly used instrument to measure the experience as well as the expression of anger (Lievaart et al., 2016).

Theoretical models explain that cognitive operations contribute substantially to a person’s level of anger experience and reactive aggression. In distinction from the emotion anger, aggression is commonly defined as a behavior or act that is intended to harm another person (DeWall et al., 2012). According to the Integrative Cognitive Model (ICM) of anger (Wilkowski and Robinson, 2008, 2010) three main cognitive processes are involved in the genesis of anger: interpretation, attention, and rumination. It is assumed that biases toward negative interpretations of social and affective information might lead to an elevated frequency of state anger and aggression. A tendency to attribute hostile traits to others is considered a central cognitive process in eliciting anger in social situations, particularly when hostile intent is ambiguous (Wilkowski and Robinson, 2010). Biases in attention are implied to enhance the interpretation-connected bias and amplify the related consequences (Wilkowski and Robinson, 2008). From the perspective of the ICM, automatic hostile interpretations should primarily contribute to individual differences in the frequency of anger elicitation (Wilkowski and Robinson, 2010). As treatment programs targeting hostile attribution biases were found to reduce anger and aggression it seems that hostile interpretation biases have causal importance in understanding individual differences in anger experience (e.g., Hudley and Graham, 1993).

In previous studies examining anger-related interpretation biases verbal descriptions of ambiguous social events were frequently administered (Orobio de Castro et al., 2002). In contrast to situations with clear negative consequences or hostile implications, which may result in similar appraisals across people events with negative as well as positive implications or intentions are better suited to assess individual differences in threat or hostile interpretation. Indicators of trait anger and aggressive personality have been found to be predictive of people’s expectations in ambiguously aggressive social situations (e.g., Epps and Kendall, 1995; Dill et al., 1997; Wenzel and Lystad, 2005). Taken together, the results of research based on verbal descriptions of social events suggest that high trait anger is associated with tendencies to interpret the behavior of others as indicating potential hostility (see for reviews Schultz et al., 2010; Owen, 2011).

In contrast, non-verbal stimuli such as faces were more rarely administered in research on anger-related interpretation biases although facial expressions of emotion are particularly salient cues for interpreting social behavior and deciding on appropriate interpersonal actions (Erickson and Schulkin, 2003; Seidel et al., 2010). In the study of Schultz et al. (2004) a combined emotion attribution score consisting of responses to facial expressions, social behaviors, and social situations was calculated. The authors observed that a greater anger attribution bias went along with more frequent experiences of anger in children. Unfortunately, the data of Schultz et al. (2004) do not allow making specific conclusions about the relationship between anger and negative interpretation of facial expressions. In their seminal study, Maoz et al. (2017) have examined the relations of trait anger, anger control and expression (as assessed by the STAXI-2) with biases in interpretation and attention in a sample of young adults. To measure interpretation bias, the authors used ambiguous facial stimuli from a morphed happy-angry continuum of images ranging from an unambiguously happy to an unambiguously angry face. Neither trait anger nor anger expression or control were related to the tendency to interpret the facial stimuli as angry. Thus, contrary to expectation, no evidence was found that trait anger is associated with hostile interpretation of ambiguous facial expressions. One improvement suggested by the authors was the inclusion of other negative emotions in addition to anger in future studies on negative interpretation bias and trait anger.

Interpretation tasks based on morphed real faces can be criticized for various reasons. Factors like cultural differences, gender or attractiveness of the encoder/actor are not taken into consideration and might have an important impact on the evaluative decisions (Hess et al., 2000; Golle et al., 2014). The perception of facial expressions (PFE) task, which consists of schematic drawings of faces (Bouhuys et al., 1995), avoids such problems. In the PFE task individuals are requested to judge how much certain emotions are represented by the schematic faces. This task has been used to measure interpretation biases in depression research (Hale, 1998; Suslow et al., 2019) and anxiety research (Bouhuys et al., 1997) but not yet in anger-related studies. Judgments of negative emotions in ambiguous but also in neutral schematic facial expressions were found to be better predictors of depressive mood and episodes than judgements of clearly negative expressions (Hale, 1998; Bouhuys et al., 1999).

Prototypical neutral faces are characterized by no facial muscle contraction (Ekman and Friesen, 1978). At first glance, one may assume that these faces appear unemotional, not conveying an affective message (Carvajal et al., 2013). However, there is evidence that people evaluate neutral faces in a negative way as sad, threatening, or cold, possibly due to the social convention to signal, at least to some extent, approval in normal interpersonal encounters (Jaeger et al., 1986; Phillips et al., 1997; Lee et al., 2008).

Previous research with real faces has also demonstrated negative distortions in the interpretation of facial expressions in anxiety and mood disorders. For example, socially anxious individuals were found to be characterized by a negative interpretative bias concerning ambiguous and neutral faces (Yoon and Zinbarg, 2008; Maoz et al., 2016; Peschard and Philippot, 2017). State anxiety in healthy individuals was also associated with the tendency to perceive negative affect in ambiguous facial expressions (Attwood et al., 2017). Negative biases in interpretation are central to cognitive theories of depression (Everaert et al., 2017). Clinical depression and dysphoric mood are both linked to negative biases in the interpretation of ambiguous and neutral facial expressions (Gollan et al., 2008; Beevers et al., 2009). Positive evaluative distortions in the perception of facial affect have been observed among individuals at risk for mania (Trevisani et al., 2008).

To our knowledge, this is the first study to examine the association of anger with negative interpretations of facial expressions controlling for anxiety and depressive mood. The current study investigated the associations between different components of anger and negative interpretation tendencies in face perception using schematic line drawings. Based on theoretical considerations (Wilkowski and Robinson, 2008, 2010) it was expected that trait anger and anger expression are both linked to heightened attribution of negative affect to ambiguous and neutral expressions. As mentioned above, ambiguous, and neutral faces seem to be the most adequate facial expressions to detect subtle negative interpretative biases. We utilized a computer-based version of the perception of facial expressions questionnaire (Bouhuys et al., 1994) to assess perceived emotions in faces. Maoz et al. (2017) administered an emotion perception task consisting of ambiguous faces mixing expressions of anger and happiness and asked their participants to decide whether the face displayed was angry or happy. Thus, Maoz et al. (2017) study focused on the perception of anger in ambiguous faces whereas in our experiment we presented features in ambiguous facial expressions, which should indicate a somewhat broader range of negative emotional states and asked participants to rate the faces concerning the expression of six basic emotions. In our study, we assessed participants’ intelligence because intelligence is a factor influencing the recognition of affects in facial expressions (Montebarocci et al., 2011). Moreover, intelligence was found to be negatively associated with anger and hostility (Zajenkowski and Zajenkowska, 2015). High levels of intelligence may help to reduce the experience of anger feelings and hostile thoughts.

## Materials and methods

### Participants

Our sample consisted of 150 healthy young individuals (75 women). The mean age of participants was 23.79 years (SD = 3.90; range: 18–35). Participants were recruited using online platforms as well as public notices posted in student halls of residence, canteens, and libraries at the University of Leipzig. School education of participants had a mean duration of 12.15 years (SD = 0.68). One hundred and nineteen participants were university students (79.33%). All participants were native speakers of German. General exclusion criteria were psychotropic medication use and actual or past presence of neurological or psychiatric diseases. The ethics committee at the Medical Faculty of the University of Leipzig approved the present study. Informed, written consent was given by all participants prior to their inclusion in the study. All participants received a financial compensation of €20.

### Questionnaires and tests

The German version of the State–Trait Anger Expression Inventory (STAXI-2; Spielberger, 1999) was administered to assess state and trait anger, and anger expression (Rohrmann et al., 2013). The state anger scale of the STAXI-2 assesses current, situational anger and consists of 15 items. The trait anger scale measures the disposition to perceive a wide range of situations as annoying and to react to these situations with state anger. The trait anger scale of the STAXI-2 comprises 10 items. The STAXI-2 has four scales assessing anger expression and anger control: Anger expression -Out (8 items) reflecting a tendency to express anger toward the environment; Anger expression -In (8 items) reflecting suppression of anger or direction of anger toward the self; Anger control -Out (5 items) referring to the ability to control and prevent anger expression toward the environment; and Anger control -In (5 items) referring to the ability to control feelings of anger by calming oneself down when angry. The items of the STAXI-2 are rated on a 4-point Likert scale (from 1 to 4). Based on the four STAXI-2 scales assessing anger expression and anger control an *anger expression index* was calculated (*cf.* Spielberger, 1999): (Anger expression - Out) + (Anger expression - In) – (Anger control - Out) – (Anger control – In) + 48. Higher anger expression index scores represent more anger expression and less control over anger experience and expression. In our study, Cronbach’s alpha was 0.86 for state anger, 0.83 for trait anger, and 0.75 for the anger expression index. Moreover, Cronbach’s alphas for the anger expression and anger control scales were 0.82 (expression – out), 0.88 (expression – in), 0.79 (control – out), and 0.79 (control – in).

The German version of the State–Trait Anxiety Inventory (Laux et al., 1981) was administered to measure participants’ state and trait anxiety. In the present sample, Cronbach’s alphas for state anxiety and for trait anxiety were 0.80 and 0.90, respectively. We used the Beck Depression Inventory (BDI-II; German version: Hautzinger et al., 2006) to assess current depressive symptoms of participants. Cronbach’s alpha was 0.87 for the BDI-II. The Mehrfachwahl-Wortschatz-Intelligenztest (MWT-B; Lehrl, 2005), a multiple-choice test using artificial and existent vocabulary of the German language, was administered to measure verbal intelligence.

### Perception of facial expressions task

**Figure 1:**
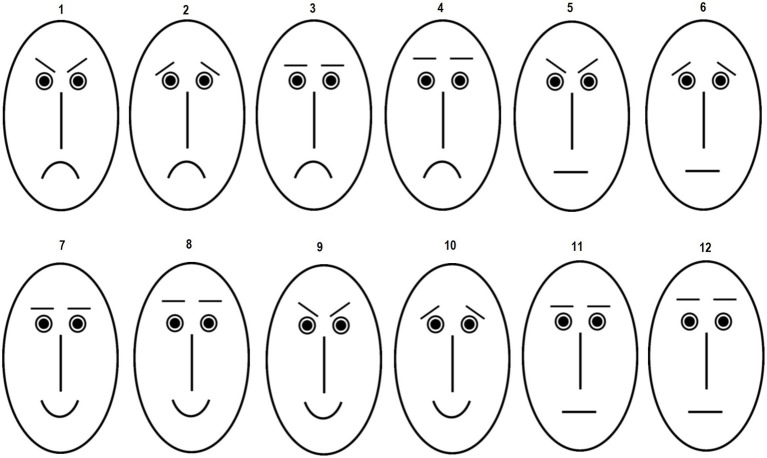
Schematic faces presented in the perception of facial expressions task. Faces 1–6 express negative affects. Faces 7 and 8 have a positive expression, faces 9 and 10 are ambiguous, and faces 11 and 12 have a neutral expression. Participants had to rate each face separately with respect to the degree to which it expresses a specific affect.

The perception of facial expressions (PFE) task comprises 12 schematic oval faces adapted from Bouhuys et al. (1994). The line drawings consist of one type of eyes and nose, three mouth and four eyebrow types (see Figure 1). Eyebrows with lowered inner corners are perceived as expressing anger and threat whereas eyebrows with elevated inner corners are evaluated as sad (Hasegawa and Unuma, 2010; Neth and Martinez, 2010). In addition, two types of horizontal eyebrows were administered, which only differed in their distance to the eyes (see Figure 1). The mouths comprised an upward-curved mouth line, a straight horizontal line, or a downward-curved mouth line. An upward-curved mouth is perceived as expressing happiness or positive mood whereas a downward-curved mouth signals negative emotion such as sadness or disgust (Wierzbicka, 1999; Zhang et al., 2021). The schematic faces can be classified as having a negative (faces 1–6), positive (faces 7 and 8), ambiguous (negative–positive (faces 9 and 10)) or neutral expression (faces 11 and 12). The line drawings were black on white background.

The program *Inquisit* (Draine, 2004) was used to present stimuli and register participants’ responses. The experiment was run on a Dell Latitude E6510 with a 15.6-inch screen. Before the start of the experiment, participants were instructed that they would see line drawings of faces. They were asked to evaluate how much the faces express sadness, anger, happiness, disgust, surprise, or anxiety. The evaluations should be made according to their subjective impression. Participants should look at each face carefully and rate each face on the degree to which it expresses a specific emotion using one of five response options (i.e., *not at all*, *a little*, *moderately*, *quite a bit* and *very much*).

Facial expressions were displayed on the left side of the screen whereas questions (e.g., “Does the face express anger?”) appeared on the right side of the screen. Faces and questions remained on the screen until an evaluative response was given. The display size of each face on the screen was about 17.3 × 10.5 cm (height by width). Responses were given on a five-point scale ranging from 1 (*not at all*) to 5 (*very much*). The response format was shown at the bottom of the screen. Participants had to rate facial expressions with regard to six basic affects (one *positive affect:* happiness; one *ambivalent affect:* surprise; and four *negative affects:* anxiety, anger, sadness, and disgust). Participants made in total 72 judgments (6 evaluative ratings × 12 faces). Responses were made on a keyboard by pressing the number keys “1,″ “2,″ “3,″ “4,″ or “5.″ Faces were presented in a random order. After a stimulus had been selected six questions were asked consecutively about its emotional expression in a fixed order: (1) Sadness, (2) Anger, (3) Happiness, (4) Disgust, (5) Surprise, and (6) Anxiety. The intertrial interval was 2 s. The distance between participants’ eyes and the screen was about 60–70 cm.

The statistical analysis of the PFE data included two steps. First, evaluation scores were computed for each affect category across all facial stimuli. Second, two negative interpretation bias scores were calculated from the evaluative data: (A) judgments of the four negative affect categories were averaged across the two ambiguous facial expressions (faces 9 and 10): negative affects in ambiguous faces; (B) judgments of the four negative categories were averaged across the two neutral facial expressions (faces 11 and 12): negative affects in neutral faces.

### General procedure

Testing sessions were conducted individually in a quiet room at the Department of Psychosomatic Medicine and Psychotherapy at the University of Leipzig. Due to the COVID-19 pandemic, all participants and the experimenter wore a face mask throughout the experiment. The tests were administered in a fixed order. After completion of the STAI-State, STAXI-2, and the STAI-Trait participants worked on the PFE task. Thereafter, participants were given the MWT-B and the BDI-II.

### Statistical analysis

Product moment correlation analysis was used to examine the relationships between anger scales, state and trait anxiety, depression, intelligence, and perception of facial expressions. To examine differences between affect conditions concerning evaluation and negative interpretation bias scores *t*-tests for dependent samples were administered. The majority of correlation analyses were conducted to control confounders and to identify variables associated with trait anger (and the anger expression index) or the negative interpretation bias scores in the PFE task. The focus of our study was on the relationships of trait anger and the anger expression index with the interpretation bias scores “negative affects in ambiguous faces” and “negative affects in neutral faces.” Hierarchical regression analyses were conducted separately for perceived negative affects in ambiguous and neutral faces to examine their relationships to trait anger (and the anger expression index), adjusting for the effects of state and trait anxiety, depression, and state anger. In general, results were considered significant at *p* < 0.05, two-tailed. All statistical calculations were made with SPSS 27.0 (IBM Corp., Armonk, NY, United States).

## Results

### Relationships between anger scales, state and trait anxiety, depression, and intelligence

First, we examined the correlations between the scales of the STAXI-2 (see Table 1 for details). State anger was positively related to trait anger and the anger expression index. Moreover, state anger was correlated with outward and inward anger expression but not with anger control. Trait anger showed high positive correlations with the anger expression index and outward anger expression as well as a high negative correlation with anger-out control (see Table 1). As could be expected, the anger expression index was positively associated with the anger expression scales and negatively related to the anger control scales.

**Table 1:** Descriptive statistics and product–moment correlations between STAXI-2 scales (*N* = 150).

	1	2	3	3a	3b	3c	Mean (SD)
1. State anger	–						15.83 (2.12)
2. Trait anger	0.26**	–					19.67 (4.51)
3. Anger expression index	0.34***	0.61***	–				45.82 (8.57)
3a. Expression-out	0.33***	0.62***	0.69***	–			11.55 (3.18)
3b. Expression-in	0.23**	0.28**	0.58***	0.12	–		16.41 (5.11)
3c. Control-out	−0.12	−0.53***	−0.63***	−0.55***	0.07	–	15.57 (2.87)
3d. Control-in	−0.10	−0.11	−0.50***	−0.18*	0.09	0.37***	14.56 (3.15)

*p ≤ 0.05; **p ≤ 0.01; ***p ≤ 0.001 (two-tailed).

Second, we examined the relations of the STAXI-2 scales with state and trait anxiety as assessed by the STAI, depression as measured by the BDI-II, and intelligence as assessed by the MWT-B. In our sample, trait anger showed small to moderate positive correlations with state and trait anxiety, and depression (see Table 2). Similar correlations were found for state anger. There were moderate to large positive correlations of the anger expression index with state and trait anxiety, and depression (see Table 2). None of the STAXI-2 scales was related to verbal intelligence.

**Table 2:** Correlations of STAXI-2 scales with state and trait anxiety (STAI), depression (BDI-II), and verbal intelligence (MWT-B) (*N* = 150).

	State anxiety	Trait anxiety	Depression	Verbal intelligence
State anger	0.27***	0.23**	0.17*	−0.12
Trait anger	0.25**	0.36***	0.19*	0.04
Anger expression index	0.28***	0.52***	0.43***	0.03
Expression-out	0.25**	0.26**	0.18*	0.03
Expression-in	0.20*	0.54***	0.46***	−0.02
Control-out	−0.16*	−0.18*	−0.14	−0.01
Control-in	−0.03	−0.11	−0.12	−0.09
Mean	35.22	39.46	8.41	109.49
SD	6.18	9.25	6.32	10.66

*p ≤ 0.05; **p ≤ 0.01; ***p ≤ 0.001 (two-tailed).

### Evaluation scores in the perception of facial expressions task: relationships with anger scales, state and trait anxiety, depression, and intelligence

According to the mean evaluation scores for the six affect categories sadness and anger were the affects most attributed to the 12 facial expressions shown in the PFE task (see Table 3). All evaluation scores differed significantly from each other (all *p*s < 0.05).

**Table 3:** Correlations of anger components (STAXI-2), state and trait anxiety (STAI), depression (BDI-II), and verbal intelligence (MWT-B) with evaluative ratings across all faces for six basic affects in the perception of facial expressions task (*N* = 150).

	Evaluative ratings					
	Anger	Anxiety	Disgust	Sadness	Surprise	Happiness
State anger	0.13	0.05	0.21*	0.11	0.23**	0.02
Trait anger	0.09	0.16	0.05	0.08	0.05	0.00
Anger expression index	0.06	0.13	0.09	0.01	0.12	0.08
Expression-out	0.11	0.09	0.17*	0.01	0.15	0.08
Expression-in	0.05	0.10	0.05	0.07	−0.05	0.09
Control-out	−0.04	−0.19*	−0.03	−0.01	−0.19*	0.05
Control-in	0.07	0.09	0.02	0.10	−0.08	−0.04
State anxiety	0.26**	0.23**	0.24**	0.12	0.16	0.02
Trait anxiety	0.06	0.10	0.09	−0.04	0.02	0.06
Depression	0.00	0.07	0.08	−0.01	0.14	0.07
Verbal intelligence	−0.15	−0.18*	−0.18*	−0.15	−0.08	−0.03
Mean	2.24	1.91	1.45	2.75	1.69	1.79
SD	0.37	0.58	0.43	0.38	0.50	0.22

*p ≤ 0.05; **p ≤ 0.01 (two-tailed).

The correlation analysis revealed that neither trait anger nor the anger expression index correlated with any of the evaluative rating scores (see Table 3). Instead, state anger was positively related to disgust and surprise ratings. Anger expression-out was positively linked with disgust evaluations and anger control-out was negatively associated with anxiety and surprise evaluations. State anxiety showed positive correlations with the perception of anger, disgust, and anxiety in the presented facial expressions. Finally, intelligence was negatively correlated with the anxiety and disgust ratings. Trait anxiety and depression were not associated with evaluative ratings in the PFE task (see Table 3).

### Negative interpretation bias scores in the perception of facial expressions task: relationships with anger scales, state and trait anxiety, depression, and intelligence

In this subsection, we tested our hypotheses that trait anger and anger expression are linked to heightened attribution of negative affect to ambiguous as well as neutral expressions. First, we examined whether the negative interpretation bias score “negative affects in neutral faces” differed from the negative interpretation bias score “negative affects in ambiguous faces.” The result of a *t*-test for dependent samples indicated that the negative interpretation bias score “negative affects in neutral faces” was significantly greater than the negative interpretation bias score “negative affects in ambiguous faces” (see Table 4), *t*(149) = 12.27, *p* < 0.001.

**Table 4:** Correlations of anger components (STAXI-2), state and trait anxiety (STAI), depression (BDI-II), and verbal intelligence (MWT-B) with negative interpretation scores in the perception of facial expressions task (*N* = 150).

	Negative affects in neutral faces	Negative affects in ambiguous faces
State anger	0.17*	0.11
Trait anger	0.23**	0.00
Anger expression index	0.16*	−0.01
Expression-out	0.15	−0.02
Expression-in	0.09	0.04
Control-out	−0.20*	0.00
Control-in	0.04	0.09
State anxiety	0.14	0.25**
Trait anxiety	0.08	0.07
Depression	0.07	0.06
Verbal intelligence	−0.19*	−0.17*
Mean	2.22	1.64
SD	0.60	0.45

*p ≤ 0.05; **p ≤ 0.01 (two-tailed).

**Figure 2:**
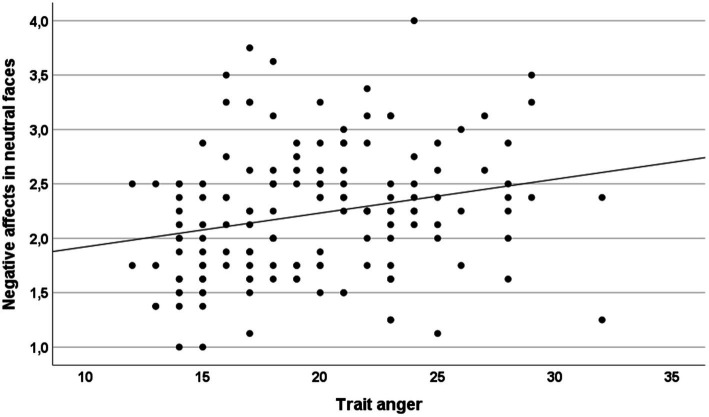
The scatterplot depicts the correlation between trait anger and the negative interpretation score “negative affects in neutral faces” (*r* = 0.23, *p* < 0.01).

According to our correlation results, trait anger, the anger expression index, and state anger were positively correlated with the interpretation bias score “negative affects in neutral faces” but not with the interpretation bias score “negative affects in ambiguous faces” (see Table 4). The correlation between trait anger and perception of negative affects in neutral faces is illustrated in Figure 2. Anger control-out was negatively related to the interpretation bias score “negative affects in neutral faces.” State anxiety was found to be positively correlated with the perception of negative affects in ambiguous faces. Finally, verbal intelligence was negatively associated with the interpretation bias scores “negative affects in neutral faces” and “negative affects in ambiguous faces” (see Table 4).

We conducted further correlation analyses to explore with which negative affect perceptions in neutral faces trait anger, the anger expression index, and state anger were associated. Trait anger showed correlations with anger (1.77, SD = 0.75), sadness (3.08, SD = 0.91), and anxiety ratings (2.40, SD = 1.02), *r* = 0.18, 0.18, and 0.19, *p*s < 0.05, but not with disgust evaluations (1.64, SD = 0.66), *r* = 0.10, *p* = 0.23. State anger was found to correlate significantly with anger and disgust evaluations, *r* = 0.19 and 0.18, *p*s < 0.05, but not with sadness and anxiety evaluations, *r* = 0.14, *p* = 0.09, and *r* = 0.04, *p* = 0.65. The correlations between the anger expression index and the four negative affect evaluation scores failed to reach statistical significance.

In addition, a regression model was calculated for the negative interpretation bias score “negative affects in neutral faces” with the predictors state and trait anxiety, depression, and state anger entered in the first step and trait anger entered in the second step. The first step did not significantly predict the negative interpretation score [*R^2^* = 0.040, *F*(4,145) = 1.52, *p* = 0.20]. The second step predicted the negative interpretation bias score [*R^2^* = 0.075, *F*(5,144) = 2.34, *p* < 0.05] with trait anger as the only significant predictor (Beta: 0.204) (see Table 5 for details).

**Table 5:** Hierarchical regression predicting the negative interpretation score “negative affects in neutral faces” as assessed in the perception of facial expressions task in two steps by state anxiety (STAI), trait anxiety (STAI), depression (BDI-II), state anger (STAXI-2), and trait anger (STAXI-2) (*N* = 150).

Coefficients						Multicollinearity		Model	
	Predictor	*β*	Beta	*t*	Sig. (*p*)	Tol.	VIF	*R* ^2^	∆*R*^2^
Step 1	State anxiety	0.010	0.100	1.05	0.30	0.73	1.38	0.040	-
	Trait anxiety	−0.001	−0.012	−0.11	0.92	0.54	1.85		
	Depression	0.002	0.022	0.21	0.83	0.63	1.58		
	State anger	0.042	0.146	1.72	0.09	0.91	1.10		
Step 2	State anxiety	0.009	0.089	0.94	0.35	0.72	1.38	0.075	0.035*
	Trait anxiety	−0.005	−0.079	−0.70	0.48	0.50	1.99		
	Depression	0.003	0.034	0.34	0.73	0.63	1.58		
	State anger	0.031	0.109	1.28	0.20	0.88	1.14		
	Trait anger	0.027	0.204	2.32*	0.02	0.83	1.20		

*β*, unstandardized regression coefficient; Tol., tolerance; VIF, variance inflation factor; *p ≤ 0.05 (two-tailed).

A second regression model was calculated for the negative interpretation bias score “negative affects in neutral faces” with the predictor anger expression index entered in the second step (instead of trait anger). In this model, the second step did not predict the negative interpretation bias score [*R^2^* = 0.051, *F*(5,144) = 1.55, *p* = 0.18], the anger expression index was a non-significant predictor (Beta: 0.127).

A *post hoc* analysis of statistical power was conducted using the program G*Power (version 3.1.9.2.; bivariate normal model) of Faul et al. (2009). To detect a small to medium effect of *r* = 0.23 with an alpha value of 0.05, one-tailed, and a sample size of 150, the achieved power is 0.886. Thus, our study had adequate power to detect small to medium associations of anger variables with negative interpretation biases.

## Discussion

The present study explored the relations between components of anger and negative interpretation tendencies in the perception of schematic facial expressions. We assumed that trait anger and anger expression are both associated with heightened attribution of negative affect to ambiguous and neutral expressions. Our analysis was focused on ambiguous, and neutral faces as they appear to be especially suited to reveal negative interpretative tendencies (Hale, 1998; Bouhuys et al., 1999). Previous investigations on anger and negative interpretation biases have used primarily verbal descriptions of ambiguous social events (Orobio de Castro et al., 2002). Our findings indicate positive relations of trait anger, anger expression, and state anger with the interpretation bias score “negative affects in neutral faces” that were of small to medium effect size. These results are consistent with our hypothesis and suggest that the tendency to experience anger in everyday life goes along with a cognitive pattern of interpreting neutral expressions in schematic faces as negative. Before generalizations on the perception of affects in neutral facial expressions can be made similar findings from additional studies are necessary, in which neutral expressions of real human faces are administered. The use of schematic faces can be criticized because of their lack of ecological validity. However, schematic facial expressions avoid problems with age, gender, or attractiveness of the encoder/actor that have shown to affect evaluations of real human faces (Hess et al., 2000; Golle et al., 2014). It should be noted that trait anger predicted the bias score “negative affects in neutral faces” after controlling for anxiety, depression, and state anger. Thus, there is evidence that trait anger could be linked to tendencies to perceive more negative affects in schematic neutral facial expressions, independent of anxiety and depressed mood. According to our findings individuals who exhibit higher levels of trait anger attribute more anger but also more sadness and anxiety to neutral faces. Interestingly, according to our data trait anger was not associated with heightened attribution of negative emotions to schematic faces in general. The relationship between trait anger and perception of negative emotions was found specifically for schematic neutral facial expressions.

In contrast, trait anger, anger expression and state anger were not correlated with the interpretation bias score “negative affects in ambiguous faces.” These results contradict our hypothesis and the assumptions made by the ICM (Wilkowski and Robinson, 2008, 2010) but interestingly they are in line with the null results of Maoz et al. (2017). It appears that trait anger is not linked to an increased attribution of anger or other negative emotions to ambiguous facial expression in samples of young adults regardless of whether the faces are presented as schematic line-drawings or morphed real faces (*cf.* Maoz et al., 2017). However, it should be noted that the schematic faces administered in our study as “ambiguous” facial expressions (see faces 9 and 10 in Figure 1) could be perceived as expressing specific affective states. These two faces were categorized as “ambiguous” because they consist of key facial features conveying conflicting signals in terms of valence, i.e., a positive (smiling) mouth and negative (“sad” or “angry”) eyebrows. However, it is possible that study participants perceived these facial stimuli not as ambiguous but as expressing specific (or clear) affective states (e.g., bravery in case of face 9 or compassion in case of face 10). The perception of a specific affective state in these faces might have made it less likely that trait angry individuals attribute negative affects to these facial expressions. To clarify the extent of ambiguity of schematic faces (and especially of faces 9 and 10) we suggest letting participants assess affective ambiguity of facial expressions in future studies. In this way, important information can be gathered concerning the subjectively perceived affective ambiguity of each facial line drawing.

Faces with a neutral expression appear, superficially considered, not to convey any affective message (Carvajal et al., 2013), but, in the last decades, evidence has accumulated that neutral faces are perceived in a negative manner as somewhat threatening or sad (Jaeger et al., 1986; Phillips et al., 1997). Lee et al. (2008) administered an implicit measure of emotional value, the Extrinsic Affective Simon Task, and observed that neutral real faces were evaluated as negative whereas ambiguous (happy–fearful) real faces were evaluated as neutral. Similarly, in our study, participants in general perceived more negative emotions in neutral than in ambiguous facial expressions. This characteristic could make the neutral condition of the PFE task more sensitive to detect negative interpretative tendencies compared to the ambiguous condition.

In the present study, participants with high trait anger attributed more anger to schematic neutral facial expression than participants with low trait anger. According to the ICM (Wilkowski and Robinson, 2008, 2010), hostile attribution represents an important cognitive process for understanding individual differences in anger experience and reactive aggression. It is assumed that trait angry individuals tend to automatically interpret ambiguous situations as hostile and attribute hostile traits to other people. These biases in turn may generate or amplify state anger and lead to more frequent elicitations of reactive aggression (Wilkowski and Robinson, 2010). It can be argued that high trait angry individuals might have more negative views and expectations of other people. These negative beliefs could have developed as a consequence of frequent experiences of hostile or frustrating behaviors from others. It is known that high levels of experienced control, inconsistency, and rejection from caregivers are accompanied by high levels of anger and hostility later in life (Meesters et al., 1995; Muris et al., 2004). Against this background, compared to low trait angry individuals, high trait angry individuals could form more negative or hostile internal models of others, which could facilitate the perception of anger and other negative affects on other people’s faces. Our work provides some evidence (for schematic neutral expressions) that high trait angry individuals attribute more anger, a hostile emotion, to others compared to low trait angry individuals, which is consistent with the ICM framework. This result is at least partially in line with evidence from a systematic review of anger bias in the perception of facial expressions across forensic, clinical, and community samples (Mellentin et al., 2015): anger-prone and aggressive populations appear to be characterized by bias toward perceiving others as angry and hostile when processing facial expressions in a variety of neuropsychological tasks. This bias seems not restricted to a deficit in selective attention but could reflect a broader bias pattern in anger-prone and aggressive individuals whereby anger and hostility are perceived in ambiguous as well as in unambiguous non-hostile facial expressions (*cf.* Mellentin et al., 2015).

Our data suggest that the negative attribution bias in trait anger may comprise not only anger but also sadness and anxiety. Facial expressions of sadness and fear are known to communicate low dominance and helplessness (Knutson, 1996; Hess et al., 2000) as well as vulnerability (Hammer and Marsh, 2015; Yeung, 2020). Thus, trait anger seems not only linked to the perception of hostility but also of weakness and low power in others. An increased disposition to develop anger experiences and reactions appears to go along with tendencies to perceive others as unfriendly or aggressive and at the same time as weak and vulnerable. The perception of weaknesses in others may facilitate the development of anger and aggression in the observer as in this case anger reactions should encounter rather favorable conditions for asserting one’s own interests. In sum, our results support the assumption that negative interpretations of others’ facial expressions in trait anger might not only include the attribution of anger but also the attribution of negative emotions related to weakness. The latter result is in line with assumptions that individuals high in trait anger should be characterized by high self-esteem, overestimation of control, power, and coping potential (Scherer and Brosch, 2009).

According to our data (schematic) neutral facial expressions could be better suited to detect negative interpretative tendencies than faces clearly expressing emotions or ambiguous faces. It appears promising to further investigate the relation between trait anger and perception of negative emotions in neutral faces by administering neutral expressions of schematic as well as real human faces in future studies and to directly compare findings between facial conditions.

In our study, participants were directly instructed to evaluate the schematic faces with respect to the degree to which they express specific affects. Thus, the observed association between negative interpretative tendencies and trait anger (for neutral expressions) was found in a task context that required participants to make explicit judgements about facial expressions. The question arises whether the manifestation of this negative interpretation bias occurs only when controlled evaluation processes are executed or if such bias may automatically emerge when confronted with neutral facial expressions. It appears, however, unlikely that in our experiment trait angry individuals were aware of their selectively increased negative interpretation of neutral faces. As pointed out above, in our study trait anger was not associated with heightened attribution of negative affects to schematic faces in general.

It is noteworthy that, in contrast to trait anger, anger expression did not significantly predict the interpretation bias score “negative affects in neutral faces” after controlling for anxiety, depression, and state anger. The anger expression index showed rather large positive correlations with trait anxiety and depressive mood in our sample. The expression of anger appears not as closely associated with negative interpretative tendencies of schematic neutral faces as trait anger, the disposition to experience and respond with anger.

In our study, we controlled the effect of other potentially relevant affective variables on negative interpretation biases such as state anxiety, trait anxiety, and depressed mood. This is an important methodological point, given that previous research using facial expressions has demonstrated that state anxiety and anxiety disorders (e.g., Maoz et al., 2016; Attwood et al., 2017) as well as clinical depression and dysphoric mood (e.g., Gollan et al., 2008; Beevers et al., 2009) can be related to negative interpretation biases. Moreover, it should be noted that we observed significant positive correlations of trait anger with state and trait anxiety and depressive mood in our study. We also assessed participants’ intelligence in our study since intelligence was found to be negatively linked to anger and hostility (Zajenkowski and Zajenkowska, 2015). In addition, intelligence seems to be a factor influencing the perception of affects in facial expressions (Montebarocci et al., 2011). However, in our study there was no evidence for an association of intelligence with state anger, trait anger, or anger expression.

The findings of our study also indicate that state anxiety was positively related to the perception of negative affects in schematic ambiguous faces. This is in line with a previous study showing a link between state anxiety and an increased attribution of anger to ambiguous faces (Attwood et al., 2017). Taken together, these data underscore that state anxiety could involve a tendency to resolve ambiguity in facial expressions in a negative manner. Results from a recent investigation (Dyer et al., 2022) indicate that things may be even more complicated than this. The authors observed greater interpretation bias toward perceiving anger in ambiguous angry–happy facial morphs during heightened state anxiety but only among individuals with high trait anxiety. Thus, it appears necessary in future research to consider the interaction of state and trait anxiety when examining facial affect processing.

It is somewhat surprising that no relations between depressed mood and negative interpretation tendencies were observed in the present study as previous research based on the PFE task has found such an association in a sample of healthy subjects (Bouhuys et al., 1995). Finally, our findings also point to a negative relation between intelligence and attribution of negative emotions to ambiguous and neutral facial expressions. This means, that intelligence might be connected to less perception of negative affect in faces with neutral or ambiguous expressions.

Treatment programs targeting hostile attribution biases and training positive interpretation were able to reduce anger and aggressive behaviors in children and adults (e.g., Hudley and Graham, 1993; Hawkins and Cougle, 2013). There are two intervention studies with computerized interpretation bias treatment that trained participants to classify ambiguous faces as happy (rather than angry). Penton-Voak et al. (2013) demonstrated that a change toward positive interpretation results in a decrease in anger and aggression in healthy adults and in adolescent youth at high risk of delinquency. In contrast, although Haller et al. (2022) observed a significant shift toward labeling ambiguous faces as happy due to a bias training they observed no improvement in irritability in youth with disruptive mood dysregulation disorder. Future intervention studies targeting hostile attribution biases in high trait anger may consider, on the one hand, the use of neutral faces as training material in addition to ambiguous faces and, on the other hand, they may not exclusively focus on the reduction of anger or hostile emotions but may also pay attention to the perception of negative emotions signaling weakness.

During the lockdowns of the COVID-19 pandemic increased anger and aggression and intensified family conflicts were observed (Agüero, 2021; Killgore et al., 2021). Within this context, the emotion of anger and its management gain new topicality. In times of the COVID-19 pandemic, wearing face masks was recommended or required in many closed public spaces, such as hospitals and public transport. Medical masks are still a part of public life in Germany. One may ask whether anger-related interpretation biases apply also (or in particular) to faces covered by masks. Emotion recognition appears to be substantially reduced in faces wearing masks (Carbon, 2020). A covered mouth region could lead to more negative interpretations of facial expressions by limiting the decoding of positive emotions like happiness (Kret and Fischer, 2018). It may be that face masks do not only negatively influence emotion recognition rates but render facial expressions highly equivocal which might then result in an elevated perception of threat (Grahlow et al., 2022). Against this background, it may be worthwhile that future studies examine the association between trait anger and the attribution of negative emotions in the perception of neutral and emotional faces wearing medical masks. Such research could help better understand whether negative or hostile interpretation tendencies may represent a cognitive factor contributing to aggressive behaviors of trait angry individuals in interactions with persons wearing face masks.

Several limitations of our study must be noted. Our sample consisted of young individuals with a mean age of nearly 24 years and a high level of education which clearly limits the generalizability of our findings. However, the mean trait anger as assessed by the STAXI-2 in our sample (19.7) was within the average range (percentile rank: 50) and study participants’ trait anger scores covered a wide range of the scale [from 12 (percentile rank: 1) to 32 (percentile rank: 97)] compared to German representative norms (age span 16–39 years; Rohrmann et al., 2013). Expanding this research beyond a college student population appears desirable, as is the examination of middle-aged and elderly adults. Previous studies explored changes in anger and aggression over the lifespan and as a function of sociodemographic variables. It was shown that the tendency to express anger and aggression decreases with age (e.g., Barefoot et al., 1993). Moreover, hostility seems to be higher in individuals with low socioeconomic status (e.g., Barefoot et al., 1991). Given the correlational nature of our data, we cannot draw any conclusions about the causal relationship between trait anger and negative interpretation bias. A further limitation of our study is the sole reliance on self-report for measuring anger experience. Future studies may include also objective measures of anger reactivity and anger expression, e.g., psychophysiological indicators such as galvanic skin response or corrugator supercilii activation during anger-provoking situations (Potegal and Qiu, 2010). In the current study, we focused exclusively on trait anger and interpretation bias. Future research may examine within persons interpretational, attentional, and rumination components of cognitive biases and their interplay in anger. However, Maoz et al. (2017) did not find evidence for an interaction effect between attentional and interpretative biases on anger measures. It should be pointed out that in cognitive bias research distortions in interpretation and attentional orientation were shown to be rather independent predictors of anxiety disorders, and depression (Pergamin-Hight et al., 2016; Klein et al., 2018). There are indications that interpretative and attentional biases can be rather uncoupled processes in healthy individuals (Todd et al., 2016a,b). In our study, facial expressions were presented on the left side of the screen. It is a limitation of our study that we did not assess handedness of our participants. The unilateral presentation of faces may have had dissimilar advantages for right-handed and left-handed individuals due to differences in hemispheric lateralization related to the processing of emotions. According to the right-hemisphere hypothesis, the right half of the brain is specialized for processing emotional information (Borod et al., 1998).

In conclusion, this is the first study to explore the association between trait anger and negative interpretation biases taking into consideration state and trait anxiety as well as depression. For schematic neutral faces, our data support an association between trait anger and negatively biased interpretation of facial expression which is independent of anxiety and depressed mood. Our data suggest that schematic neutral facial expressions might be useful stimuli in the future study of anger-related interpretation biases. The negative interpretations of other people’s facial expressions observed in trait angry individuals could not only comprise the attribution of anger but also the attribution of negative emotions signaling weakness.

## Data availability statement

The raw data supporting the conclusions of this article will be made available by the authors, without undue reservation.

## Ethics statement

The studies involving human participants were reviewed and approved by Ethik-Kommission der Medizinischen Fakultät, Universität Leipzig. The patients/participants provided their written informed consent to participate in this study.

## Author contributions

TS conceived and designed the experiment with contributions from AK. PR was engaged in data collection. PR analyzed the data under the supervision of TS. PR wrote the manuscript with revisions and contributions from TS and AK. All authors contributed to the article and approved the submitted version.

## Funding

The authors acknowledge support from the German Research Foundation (DFG) and Universität Leipzig within the program of Open Access Publishing.

## Conflict of interest

The authors declare that the research was conducted in the absence of any commercial or financial relationships that could be construed as a potential conflict of interest.

## Publisher’s note

All claims expressed in this article are solely those of the authors and do not necessarily represent those of their affiliated organizations, or those of the publisher, the editors and the reviewers. Any product that may be evaluated in this article, or claim that may be made by its manufacturer, is not guaranteed or endorsed by the publisher.
